# Frequency and Predictors of Relapses following SARS-CoV-2 Vaccination in Patients with Multiple Sclerosis: Interim Results from a Longitudinal Observational Study

**DOI:** 10.3390/jcm12113640

**Published:** 2023-05-24

**Authors:** Niklas Frahm, Firas Fneish, David Ellenberger, Judith Haas, Micha Löbermann, Melanie Peters, Dieter Pöhlau, Anna-Lena Röper, Sarah Schilling, Alexander Stahmann, Herbert Temmes, Friedemann Paul, Uwe Klaus Zettl

**Affiliations:** 1 MS Forschungs- und Projektentwicklungs-gGmbH (MS Research and Project Development gGmbH [MSFP]), 30171 Hannover, Germany; fneish@msregister.de (F.F.); ellenberger@msregister.de (D.E.); peters@msregister.de (M.P.); roeper@msregister.de (A.-L.R.); schilling@msregister.de (S.S.); stahmann@msregister.de (A.S.); 2 Department of Neurology, Neuroimmunological Section, University Medical Center of Rostock, 18147 Rostock, Germany; uwe.zettl@med.uni-rostock.de; 3 Deutsche Multiple Sklerose Gesellschaft, Bundesverband e.V. (German MS Society Federal Association [DMSG]), 30171 Hannover, Germany; haas-heide@gmx.de (J.H.); dieter.poehlau@kamillus-klinik.de (D.P.); temmes@dmsg.de (H.T.); 4 Department of Tropical Medicine, Infectious Diseases and Nephrology, University Medical Center of Rostock, 18057 Rostock, Germany; micha.loebermann@med.uni-rostock.de; 5 Gesellschaft für Versorgungsforschung mbH (Society for Health Care Research [GfV]), 30171 Hannover, Germany; 6 Experimental and Clinical Research Center, a Joint Cooperation between the Max Delbrück Center for Molecular Medicine in the Helmholtz Association and the Charité Medical Faculty, Campus Berlin-Buch, 13125 Berlin, Germany; friedemann.paul@charite.de; 7 Department of Neurology, Charité—Universitätsmedizin, 10117 Berlin, Germany; 8 NeuroCure Clinical Research Center, Charité—Universitätsmedizin, 10117 Berlin, Germany

**Keywords:** SARS-CoV-2, COVID-19, vaccination, multiple sclerosis, relapse

## Abstract

Despite protection from severe COVID-19 courses through vaccinations, some people with multiple sclerosis (PwMS) are vaccination-hesitant due to fear of post-vaccination side effects/increased disease activity. The aim was to reveal the frequency and predictors of post-SARS-CoV-2-vaccination relapses in PwMS. This prospective, observational study was conducted as a longitudinal Germany-wide online survey (baseline survey and two follow-ups). Inclusion criteria were age ≥18 years, MS diagnosis, and ≥1 SARS-CoV-2 vaccination. Patient-reported data included socio-demographics, MS-related data, and post-vaccination phenomena. Annualized relapse rates (ARRs) of the study cohort and reference cohorts from the German MS Registry were compared pre- and post-vaccination. Post-vaccination relapses were reported by 9.3% PwMS (247/2661). The study cohort’s post-vaccination ARR was 0.189 (95% CI: 0.167–0.213). The ARR of a matched unvaccinated reference group from 2020 was 0.147 (0.129–0.167). Another reference cohort of vaccinated PwMS showed no indication of increased post-vaccination relapse activity (0.116; 0.088–0.151) compared to pre-vaccination (0.109; 0.084–0.138). Predictors of post-vaccination relapses (study cohort) were missing immunotherapy (OR = 2.09; 1.55–2.79; *p* < 0.001) and shorter time from the last pre-vaccination relapse to the first vaccination (OR = 0.87; 0.83–0.91; *p* < 0.001). Data on disease activity of the study cohort in the temporal context are expected for the third follow-up.

## 1. Introduction

The global COVID-19 pandemic caused anxiety among people with multiple sclerosis (PwMS) regarding the risk of a severe COVID-19 disease after infection with severe acute respiratory syndrome coronavirus type 2 (SARS-CoV-2), especially when the immune response may be compromised by immunotherapies. Although the overall risk of SARS-CoV-2 infection does not seem to be increased in PwMS, recent studies point to a greater risk of SARS-CoV-2 infections and a severe COVID-19 disease in PwMS with comorbidities, higher neurological disability, and treatment with B-cell-depleting drugs [1,2,3,4,5,6,7].

Several vaccines against COVID-19 have been approved since summer 2020, and an unambiguous recommendation for all PwMS to be vaccinated has been strongly advocated by expert organizations all over the world ever since [8]. This recommendation stands although PwMS were not enrolled in the pivotal vaccination trials and despite observations of compromised humoral immunity following SARS-CoV-2 vaccines in patients on B-cell-depleting therapies or sphingosine-1-phosphate (S1P) receptor modulators [9,10,11,12], given that sufficient T cell vaccine responses may, nonetheless, confer protection against severe COVID-19 disease courses [13,14].

However, as with previous vaccines, there is general skepticism towards the efficacy, tolerability, and harmlessness of SARS-CoV-2 vaccines in parts of the population, and especially among PwMS [15,16,17]. Single reports on demyelinating events following these vaccinations stirred concerns that relapses may be triggered by vaccinations, attack rates might be increased, or pre-existing symptoms could be worsened in temporal association with vaccination dates. However, as single demyelinating events in temporal proximity to SARS-CoV-2 vaccination never allow any conclusions regarding causality, a robust analysis of the interaction of SARS-CoV-2 vaccines with the MS disease course and immunotherapy is of paramount importance. This may help empower physicians to adequately address vaccine hesitancy in PwMS [18] and provide sound counselling.

Previous studies on relapse rates, side effects, and tolerability following SARS-CoV-2 vaccinations were limited by small sample sizes, a single-centre approach, and the provision of data on only one of several available vaccines [19,20,21]. There was only one large study examining and comparing over 6000 PwMS from Germany and the United Kingdom (UK), but with a focus on SARS-CoV-2 vaccine reactions rather than vaccine-associated relapses [22]. Therefore, for a better understanding of tentative associations between SARS-CoV-2 vaccines and disease activity in PwMS, it is indispensable to investigate larger populations that are representative of the demographic and clinical spectrum of MS including the use of disease-modifying drugs (DMDs), and to analyze all available vaccines. Against this background, we aimed to address the following questions: (1) How many PwMS reported MS relapses before and after vaccinations against SARS-CoV-2, depending on vaccines administered and immunotherapy used? (2) What predictors for the occurrence of post-vaccination relapses exist?

## 2. Materials and Methods

**Figure 1:**
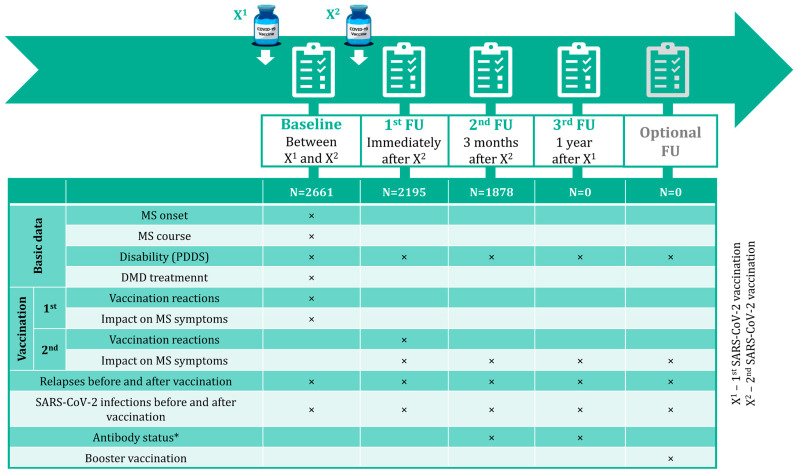
Study design and data collection. Four patient-reported surveys were conducted within one year after the first SARS-CoV-2 vaccination of PwMS: a baseline survey after the first and before the second vaccination, the first follow-up immediately after the second vaccination (in the case of two-dose vaccines), the second follow-up three months after the second vaccination/completed basic immunization, and the third follow-up one year after the first vaccination. Furthermore, there is a possibility to conduct an optional follow-up to analyze specific subgroups of PwMS. Across the surveys, data on demographics, MS disease status, MS therapy, and SARS-CoV-2 vaccination were acquired. The current analysis covers data from the baseline, the first follow-up as well as the second follow-up up to and including 3 November 2021. DMD—disease-modifying drug; MS—multiple sclerosis; N—number of patients; PDDS—patient-determined disease steps; PwMS—people with MS; SARS-CoV-2—severe acute respiratory syndrome coronavirus 2; X^1^—first SARS-CoV-2 vaccination; X^2^—second SARS-CoV-2 vaccination; *—patient-reported antibody detection (yes/no).

The longitudinal Germany-wide online survey included people with the following (patient-reported) exposure criteria: MS diagnosis, ≥18 years old, administration of ≥1 SARS-CoV-2 vaccine dose approved by the European Medicines Agency, and electronically consented to participate in the survey voluntarily. Data collection during this prospective, non-interventional, observational study was conducted through patient-reported online questionnaires on the website of the German Multiple Sclerosis Society (Deutsche Multiple Sklerose Gesellschaft or DMSG). Participation in the study was advertised via social media channels and the homepage of the DMSG and directly via MS centres participating in the German MS Registry (GMSR). A baseline survey and three follow-ups were planned (see Figure 1). Starting from 3 May 2021, PwMS who fulfilled the inclusion criteria had the opportunity to participate in the baseline survey (BS; after registration via the DMSG website). After these participants should have received their second SARS-CoV-2 vaccination (X^2^; for two-dose vaccines) according to the recommendations of the German Standing Committee on Vaccination (STIKO), they were invited to complete the first follow-up (FU1). These patients were asked to participate in another (second) follow-up (FU2; three months after completing the vaccination/receiving the second dose), and in a final (third) follow-up (FU3; one year after the first vaccination). Moreover, an additional as well as optional survey among specific patient subgroups within the one-year period was anticipated to cover “booster” vaccinations. Data on demographics (e.g., gender, age), clinical MS details (e.g., MS onset date, disability level [patient-determined disease steps or PDDS], MS course, DMDs used), and SARS-CoV-2 vaccinations (e.g., vaccines administered, occurring vaccination reactions, patient-reported MS relapses before [date of the latest relapse prior to the initial vaccination] and after vaccinations [number of relapses from the first vaccination [X^1^] to FU2, including a control question for diagnosed relapses only during FU2]) were collected across the surveys; see Figure 1. This analysis comprises BS, FU1, and FU2. Power calculations assuming a probability difference as effect size as small as 0.06 result in a sample size of 2180 given a power of 80% and a type-I-error of 5%. Online questionnaires are provided in the Supplementary Documents S1–S3.

### Statistical Analysis

Data export for this analysis was on 3 November 2021. The study cohort was characterized by calculation of means, standard deviations, medians, and percentages. Patient subgroups were compared using chi-square test, Fisher’s exact test, Kruskal–Wallis rank-sum test and Mann–Whitney U test. Significance level was set at α = 0.05. To reveal predictors for the presence of post-vaccination relapses, univariable and multivariable logistic regression models were fitted to examine age, gender, disability level, DMD treatment status, disease duration, and time from the last relapse (before X^1^) to X^1^. The extrapolated annualized relapse rates (ARRs) and 95% confidence intervals (CIs) were calculated for the study cohort in total and for patients stratified by vaccine administered and DMD group used. The term “extrapolated ARR” was used to describe that the ARR might be estimated on patients without a full year of observation time. To validate the quality of our patient-reported relapse data, we additionally calculated the ARR of two reference cohorts from the GMSR. On the one hand, we analyzed a cohort of SARS-CoV-2-vaccinated registry patients to obtain ARR pre- (2020) and post-vaccination (2021) (vaccination data are only available for a subgroup; date of GMSR data export: 14 November 2022). On the other hand, we calculated the pre-pandemic ARR (2020) of a reference registry cohort, which was comprehensively matched to the survey cohort. Gender, DMD treatment (high-efficacy/mild–moderate-efficacy/untreated) [23], MS disease course, disease duration, age at MS onset, time to diagnosis, and disability level were used as variables in a 1:1 multivariable matching according to Hansen et al. [24]. In the GMSR reference groups, relapses were clinically confirmed by the documenting MS centres. Further information on GMSR data acquisition and infrastructure are provided in the article by Ohle et al. [25]. Statistical analyses, data transformation, and the generation of figures were performed using R 4.0 (The R Foundation for Statistical Computing, Vienna, Austria).

## 3. Results

### 3.1. Study Population and SARS-CoV-2 Vaccines Administered

Until the data export date (3 November 2021), 2661 PwMS participated in the BS (2583 completed). Of those patients, 2195 participated in FU1 and 1878 in FU2 (2185 and 1867 completed, respectively). The three related patient cohorts were characterized and compared demographically and clinically (see Table 1). Except for the disability level (chi-square test [baseline vs. FU1]: *p* = 0.019), there were no significant differences between the survey cohorts (*p* ≥ 0.073). The median time from X^1^ to the completion of the BS is 1.6 months (25% quantile: 0.7 months, 75% quantile: 2.8 months), from X^2^ to FU1 0.8 (0.5, 1.8) months, and from X^1^ to FU2 4.5 (4.3, 4.9) months. A total of 783 participants were lost from BS to FU2, of which 577 had no specific reason for non-participation (Supplementary Table S1).

Two doses of tozinameran were the most frequently administered vaccination scheme, followed by two doses of elasomeran and two doses of different vaccines (heterologous); see Table 2. Detailed information on the vaccine distribution over time, the frequency of SARS-CoV-2 infections following vaccinations, and the reported vaccination reactions are shown in the Supplementary Documents S4–S6.

### 3.2. Relapses before and after SARS-CoV-2 Vaccination in the Study Population

Patients with relapses within the year prior to X^1^ (N = 391, 14.7%) were most frequently treated with cladribine, dimethyl fumarate, or teriflunomide (27.4%), followed by (peg-) interferon beta or glatiramer acetate (19.2%), anti-CD20 antibodies (11.5%), S1P receptor modulators (9.2%), natalizumab (5.1%), and others (1.5%). About a quarter of these 391 patients were DMD-untreated at the time of BS (24.0%). During BS and FU1, 193 patients indicated post-vaccination relapses (7.3%; exclusively after X^1^: N = 95, exclusively after X^2^: N = 81, after both: N = 17). Of those patients, 86 (44.6%) received corticosteroids as relapse treatment: 29 after X^1^ (33.7%), 48 after X^2^ (55.8%), and 9 after both vaccinations (10.5%). FU2 reveals 135 patients reporting relapses after X^2^/latest vaccination (81 patients with previously reported relapses [in BS or FU1] and 54 patients with newly reported relapses), with 106 of them (78.5%) reporting that the relapses were also diagnosed by the treating neurologist. In total, relapses from X^1^ to FU2 with a median observation period of 4.5 months were recorded in 247 PwMS. Patients with post-vaccination relapses were younger, had a diagnosis of relapsing–remitting MS more often, were less frequently DMD-treated, reported relapses more often within the year prior to X^1^, and had a shorter time from the latest relapse (before X^1^) to X^1^; see Table 3.

**Figure 2:**
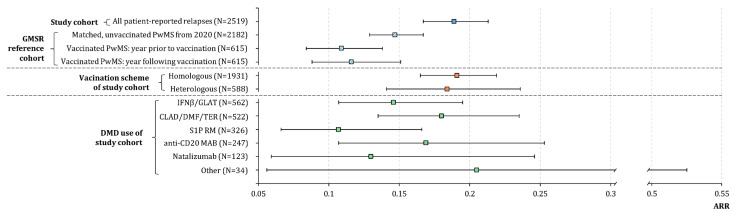
(Extrapolated) annualized relapse rates following SARS-CoV-2 vaccinations in patients with multiple sclerosis stratified by vaccination scheme and disease-modifying drug use. The ARRs are visualized as boxes parallel to the *x*-axis, flanked by whiskers that define the 95% confidence interval. The ARR of the study cohort is 0.189 (95% CI: 0.167–0.213). Comparing ARRs of a smaller GMSR reference group in the year before vaccination with the year following vaccination reveals no significant difference (0.109 [95% CI: 0.084–0.138] vs. 0.116 [95% CI: 0.088–0.151]). Patients with a homologous vaccination scheme (total: 0.191 [95% CI: 0.165–0.219]│solely tozinameran: 0.180 [95% CI: 0.154–0.210]│solely elasomeran: 0.283 [95% CI: 0.195–0.398]│solely AZD1222: 0.161 [95% CI: 0.065–0.332]│insufficient patient number for Ad26.COV2.S [N = 22]) show a similar disease activity compared to those with a heterologous scheme (0.184; 95% CI: 0.141–0.236). Considering the different DMD groups, patients with CLAD/DMF/TER reveal the highest extrapolated ARR (0.180; 95% CI: 0.135–0.235), followed by those with anti-CD20 MAB (0.169; 95% CI: 0.107–0.253), IFNβ/GLAT (0.146; 95% CI: 0.107–0.195), natalizumab (0.130; 95% CI: 0.059–0.246), and S1P RM (0.107; 95% CI: 0.066–0.166). Patients with other DMDs (N = 31) have an extrapolated ARR of 0.205 but show a 95% CI of 0.056–0.525. anti-CD20 MAB—anti-CD 20 monoclonal antibody: ocrelizumab/ofatumumab/rituximab; ARR—annualized relapse rate; CLAD/DMF/TER—cladribine/dimethyl fumarate/teriflunomide; CI—confidence interval; DMD—disease-modifying drug; IFNβ/GLAT—interferon beta-1a/interferon beta-1b/peginterferon beta-1a/glatiramer acetate; MS—multiple sclerosis; N—number of patients; PwMS—people with MS; S1P RM—sphingosin-1-phosphate receptor modulator: fingolimod/ozanimod/siponimod; SARS-CoV-2—severe acute respiratory syndrome coronavirus 2.

The extrapolated post-vaccination ARR of the PwMS analyzed (N = 2519, without patients with primary progressive MS [PPMS]) with a median observation period of 4.5 months was 0.189 (95% CI: 0.167–0.213); see Figure 2. Moreover, we matched a pre-pandemic, unvaccinated GMSR reference group of 2182 PwMS from 2020 (median observation period: 9.1 months; Supplementary Table S2) to our study cohort, resulting in an ARR of 0.148 (95% CI: 0.129–0.168). To validate the patient-reported relapse data of our study cohort, we also considered a smaller reference cohort of vaccinated PwMS from the GMSR (N = 615); see Supplementary Table S3. The ARR of this reference group does not change significantly when comparing the year before vaccination (0.109; 95% CI: 0.084–0.138) and the year following vaccination (0.116; 95% CI: 0.088–0.151). The ARR stratified by vaccination scheme and DMD groups used is shown in Figure 2.

**Figure 3:**
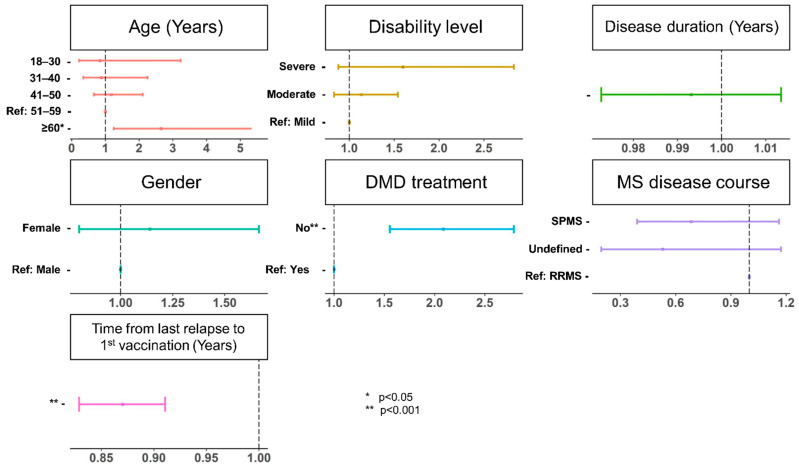
Patient characteristics associated with the occurrence of relapses in a multivariable logistic regression model. The odds ratios are displayed as dots parallel to the *x*-axis, flanked by whiskers that define the 95% confidence interval. The absence of DMD treatment, the time from the last pre-vaccination relapse to the first vaccination, and an age ≥ 60 years (reference: 51–59 years) are significantly associated with the presence of post-vaccination relapse (*p* ≤ 0.031). Age as a numerical variable reveals no significant association (*p* = 0.448). DMD—disease-modifying drug; MS—multiple sclerosis; *p*—*p*-value; ref—reference; RRMS—relapsing-remitting MS; SPMS—secondary progressive MS.

In the multivariable model, the absence of DMD treatment (OR = 2.09, 95% CI: 1.55–2.79, *p* < 0.001) and the time from the last pre-vaccination relapse to X^1^ (OR = 0.87, 95% CI: 0.83–0.91, *p* < 0.001) are significantly associated with relapses following vaccination; see Figure 3. Age also emerges as a predictor of post-vaccination relapse, but only in the age group ≥ 60 years (OR = 2.65, 95% CI: 1.25–5.55, *p* = 0.031, reference: 51–59 years). When age is considered as a numerical variable, no significant association is observed (OR = 0.98, 95% CI: 0.93–1.03, *p* = 0.448). Results of the univariable regression model are shown in Supplementary Figure S1.

## 4. Discussion

Due to the design of pivotal vaccination trials against SARS-CoV-2 that explicitly exclude patients with autoimmune diseases such as MS [26,27,28,29,30], the entirely new biphasic nucleic acid-based vaccines (mRNA- and vector-based vaccines) [31,32,33] and the limited initial results on the efficacy and side effect spectrum of these vaccines after approval in patients with autoimmune diseases, results from large multi-centre studies in real-world settings are urgently needed for daily clinical practice [34,35].

Since 26 December 2020, vaccinations against SARS-CoV-2 have been carried out in Germany [36]. As the initial quantity of vaccine doses was insufficient to meet the entire demand of Germany, priority risk groups were defined by the Robert Koch Institute [37], rendering PwMS eligible to be vaccinated from late spring 2021 onwards. In terms of vaccine distribution, differences occur between the PwMS in our study and the general German population. Study participants report more frequent administration of mRNA vaccines (tozinameran, elasomeran) than indicated in the total German population (X^1^: 88.0% vs. 78.3%; X^2^: 96.5% vs. 93.4%; Fisher’s exact test: *p* < 0.001, respectively) [38]. However, the trend in the study population and the general German population is consistent that mRNA vaccines are administered more frequently than vector vaccines, with Ad26.COV2.S as the least frequently used vaccine.

The present study took advantage of the established infrastructure of the DMSG, which enabled outreach to numerous PwMS across the country within a short period of time. By employing a structured survey with 2661 respondents on the relapse activity in temporal association with all four then-approved SARS-CoV-2 vaccines, we were able to overcome some limitations of previous studies that had smaller sample sizes, a monocentric design, or reported data on only one of the available vaccines [19,20,21]. In a previous study that focused on post-vaccination side effects, over 6000 PwMS from Germany and the UK were analyzed [22]. In fact, relapse data were also presented in the German/UK paper, but those data were only available for 2346 German patients (relapses after X^1^/X^2^ in 6% of patients). Furthermore, the median observation period of the German patients was only two months since X^1^. Thus, the enrolment of more than 2600 participants of the present study with a median observation period since X^1^ of 4.5 months (by inclusion of FU2) yielded sufficient statistical power to perform univariable as well as multivariable analyses on the predictive factors of relapse activity following vaccination. Additionally, the survey patient cohort is representative for PwMS in Germany regarding socio-demographic and clinical composition (Supplementary Document S7).

From a clinical and counselling perspective, our data on post-vaccination relapse rates are probably the most relevant results of this study. Not surprisingly, the PwMS who report post-vaccination relapses are younger in mean, more often have an RRMS course, are less frequently treated with DMDs, and more often had relapses in the year prior to X^1^, compared with PwMS who do not report post-vaccination relapses (Table 3). However, mainly the absence of DMD treatment and the time from the last pre-vaccination relapse to X^1^ are found to be predictors for the occurrence of post-vaccination relapses. The seemingly lower relapse numbers in the period following the vaccinations (9.3%) compared with the year prior to X^1^ in our survey population (14.7%) should not be overinterpreted because multiple causes might apply, such as regression to mean, reporting bias, and others. Moreover, it is unclear to what extent government measures imposed to contain the pandemic, such as face coverings, social distancing, cancellations of mass events, etc., might have impacted relapse rates. Although the extrapolated ARR of the survey cohort is slightly higher than that of the unvaccinated pre-pandemic registry reference cohort (albeit with overlapping 95% CIs), no direct conclusions can be drawn from this. A direct comparison between survey and matched reference cohort is prone to bias due to different data acquisition strategies (patient- vs. physician-reported). To validate our findings, we considered the relapse activity of another smaller reference cohort of vaccinated PwMS from the GMSR (N = 615). We compared the ARR of this vaccinated reference cohort in the year prior to SARS-CoV-2 vaccination with the year following vaccination to validate the quality of our patient-reported relapse data. The resulting ARR of 0.116 is considerably lower than the rate of our survey cohort (0.189). This difference can be partially—we hypothesize for about 0.03—attributed to the patient-reported data collection of the study cohort (compared with clinically confirmed relapses of the reference cohort). For about 0.04, the difference may be attributed to a cohort effect, i.e., DMD treatment rates are much higher in registry patients (91%) than in the survey participant/matched cohort (72%). The salient finding regarding the vaccinated reference cohort from the GMSR is the absence of a signal suggesting a significantly increased relapse rate or triggering of relapse activity following SARS-CoV-2 vaccination (year before vaccination: 0.108, year following vaccination: 0.116). Also for other autoimmune diseases, e.g., those affecting the thyroid, the lung or the blood, there is no clear opinion on a well-founded association between SARS-CoV-2 vaccination and subsequent disease exacerbation. Considering the analysis of the post-vaccination relapse activity in PwMS in dependence of the vaccine type administered or the DMD used, a case-report-based systematic review by Nabizadeh et al. identified 29 cases of relapse, which occurred, on average, 9.5 days after SARS-CoV-2 vaccination in PwMS [39]. Among these 29 PwMS, tozinameran and AZD1222 (N = 12, 41.4%, respectively) were the most commonly administered vaccines. Possible trigger mechanisms for relapses due to certain vaccines are discussed, e.g., cross-reactivity by mRNA-based vaccines (structural similarity between SARS-CoV-2 spike protein antibody and myelin basic protein) [40] and bystander activation by vector-based vaccines (induction of inflammatory processes by adjuvants) [41,42]. Dimethyl fumarate (N = 6, 20.7%), fingolimod (N = 3, 10.3%), and interferon beta (N = 3, 10.3%) were used by most of the 29 PwMS with relapses in the study by Nabizadeh et al. However, the DMDs used were not reported for 9 of 29 PwMS (31.0%) [39]. Our results suggest slightly different ARRs between patients with different DMDs, but with overlapping confidence intervals. Moreover, in the cross-sectional observational study by Alroughani et al., in which 647 PwMS were evaluated for SARS-CoV-2 vaccine safety, the use of DMDs was not associated with worsening MS symptoms or the occurrence of post-vaccination relapses [43]. Therefore, to prevent post-vaccination relapses, attention should be paid to adequate treatment with DMDs, as our analyses show that the absence of DMDs is a major risk factor for relapses in PwMS. Generally, the key issue is that post-vaccination disease exacerbations are often discussed in single or small collections of case reports [44,45,46,47], but studies with larger study populations and the associated power are scarce. In light of single case reports describing demyelinating events in temporal association with the SARS-CoV-2 vaccines and numerous unsubstantiated assertions disseminated by social media on a causal association of vaccinations against COVID-19 and adverse medical outcomes, our data are reassuring for PwMS, their caregivers, and treating neurologists.

Our study has several limitations. We tried to raise awareness of the study among many participants through widespread advertising in different channels. Nevertheless, there is a risk of bias, as not all vaccinated PwMS want or are able to participate in an online survey. However, we endeavored to reach as many patients as possible, e.g., by social media channels, homepage, and participating centres. Furthermore, MS relapses and SARS-CoV-2 breakthrough infections were exclusively patient-reported and not objectively confirmed by medical records or treating neurologists, while relapses of the reference cohorts from the GMSR were clinically confirmed by MS centres. As the data were extracted from the survey, we have no confirmed information on immune responses to the SARS-CoV-2 vaccines among the study participants. This also limits the explanatory power of these data to a certain degree. Nevertheless, a control question was included in FU2 asking which of the reported relapses had also been diagnosed by a physician. Unfortunately, data on clinically confirmed relapses were not available for BS and FU1 and, thus, are a relevant limitation of this study. We, therefore, cannot fully rule out that pseudo-exacerbations, such as the so-called Uhthoff phenomena [48], were reported as relapses. It is, however, unconceivable that this was a massive confounder of the principal findings on post-vaccination relapse rates: slightly below 50% of patients report relapse treatment with corticosteroids, suggesting that the neurological deficits are adjudicated by neurologists as severe enough to indicate relapse treatment. The fact that less than half of patients report relapse treatment with corticosteroids does also not automatically suggest a massive influence of pseudo-relapses/patients reporting Uhthoff’s phenomena: according to the German guidelines, the indication for corticosteroid treatment should be established in light of relapse severity, functional deficits, and impact of quality of life, which would result in a proportion of milder relapses without treatment [23]. It is conceivable that many neurologists were hesitant to treat any relapse regardless of clinical severity with corticosteroids during the pandemic, presumably owing to concerns that high dose corticosteroids might lower vaccine efficacy [49]. Nevertheless, our comparisons to registry data suggest that pseudo-relapses partially affect self-reported ARR, but only to a moderate extent, likely to be less than 20% overestimation of clinically assessed ARR. We expect data on the clinically confirmed relapses since X^1^ for the one-year survey (third follow-up). Data on post-vaccination relapses were gathered mostly over the summer and early autumn months, thereby potentially over- or underestimating the relapse rates over a full 12 month cycle due to seasonal variation in relapse activity [50,51]. Another limitation is the observation period of the study cohort of less than six months. Thus, we were only able to provide extrapolated ARRs for the study cohort, unlike the reference cohorts. Further, the time from vaccination to the relapse was not acquired. The one-year observation period and questions on the period from vaccination to the occurrence of subsequent relapse were included in FU3 and could provide further information on short-term or long-term relapse activity following SARS-CoV-2 vaccination. Those data are expected this year (FU3). Moreover, information on the booster vaccine is not yet available. Another drawback of the survey study is that pre-vaccination relapse data are not reliably assessable. Only data on the last relapse before X^1^ were collected, not the number of relapses in the year before X^1^. In conclusion, the pre-vaccination ARR was not included in the study plan. For comparisons we, therefore, needed to use an unvaccinated reference cohort (2020) as matched controls from a different data source. For the smaller vaccinated registry cohort, we could, however, use pre- and post-vaccination data to conduct self-controlled case series analyses [52,53,54]. Finally, one third of the cohort is lost to follow-up, which, in addition with a relatively short observation interval, limits conclusions on relapses and infections following vaccination and, thus, biasing longitudinal results. Therefore, this is an interim analysis. A complete perspective with increased statistical power is expected when we acquired the one-year data after initial SARS-CoV-2 vaccination. Nevertheless, this is one of the largest representative samples of PwMS to date that provides results of paramount importance on the association of relapse activity with SARS-CoV-2 vaccines. Based on the available clinical data, PwMS are strongly advised to be vaccinated against COVID-19, more so because it seems to confer some protection against severe COVID-19 courses related to the emerging SARS-CoV-2 variants of concern [55].

## 5. Conclusions

In conclusion, our prospective non-interventional observational study comprises one of the largest datasets concerning information on SARS-CoV-2 vaccinations in PwMS. During a median observation period of 4.5 months following vaccination, relapses occur in 9% of patients (extrapolated ARR of 0.19) analyzed and are mainly associated with the absence of a DMD treatment and a shorter time from the last relapse to X^1^. A comparison of ARRs one year before and after vaccination in a reference cohort from the GMSR also indicates no substantially increased short-term relapse activity after SARS-CoV-2 vaccination in PwMS, which is similar to other widely recommended vaccines, e.g., against influenza, or poliomyelitis. For long-term evaluations regarding relapse activity after SARS-CoV-2 vaccination in our survey cohort, data with a minimum observation period of one year are required, which are expected this year. The investigation of vaccination breakthroughs and possibly associated long-COVID and post-COVID diseases is also an important perspective for the future.

## Figures and Tables

**Table 1:** Study population.

	Baseline (N = 2661)	FU1 (N = 2195)	FU2 (N = 1878)
Gender, N (%)			
Female	2058 (77.9)	1726 (79.3)	1464 (78.5)
Male	574 (21.7)	444 (20.4)	394 (21.1)
Divers	9 (0.3)	7 (0.3)	6 (0.3)
Age [years], median (range)	45.2 (18.0–83.8)	45.5 (18.0–81.0)	46.7 (18.1–83.8)
MS disease course, N (%)			
RRMS	1987 (74.7)	1656 (75.4)	1405 (74.8)
SPMS	456 (17.1)	368 (16.8)	326 (17.4)
PPMS	102 (3.8)	78 (3.6)	70 (3.7)
Undefined	116 (4.4)	93 (4.2)	77 (4.1)
Disability level (PDDS), N (%)			
Mild (0–1)	1376 (51.7)	1194 (54.6)	979 (52.4)
Moderate (2–4)	965 (36.3)	735 (33.6)	664 (35.6)
Severe (≥5)	320 (12.0)	256 (11.7)	224 (12.0)
Coincident autoimmune diseases, N (%)	572 (21.5)	479 (21.8)	396 (21.1)
DMD treatment, N (%)	1921 (72.2)	1603 (73.1)	1392 (74.1)
IFNβ/GLAT	571 (30.4 ^a^)	488 (31.1 ^b^)	418 (30.7 ^c^)
CLAD/DMF/TER	533 (28.4 ^a^)	451 (28.8 ^b^)	391 (28.8 ^c^)
S1P RM	333 (17.7 ^a^)	275 (17.5 ^b^)	248 (18.2 ^c^)
anti-CD20 MAB	287 (15.3 ^a^)	222 (14.2 ^b^)	187 (13.8 ^c^)
Natalizumab	125 (6.6 ^a^)	104 (6.6 ^b^)	91 (6.7 ^c^)
Other	31 (1.6 ^a^)	28 (1.8 ^b^)	25 (1.8 ^c^)
Relapse within the year prior to X^1^, N (%)	391 (14.7)	315 (14.4)	262 (14.0)
Relapse within 6 months prior to X^1^, N (%)	213 (8.0)	169 (7.7)	139 (7.4)
Relapse within 3 months prior to X^1^, N (%)	100 (3.8)	77 (3.5)	60 (3.2)
Time from last relapse (before X^1^) to X^1^ [years], median (range)	3.1 (0.03–40.7)	3.2 (0.03–40.7)	3.2 (0.03–40.7)

^a^—referring to 1880 patients with detailed data on the DMD used (baseline); anti-CD20 MAB—anti-CD 20 monoclonal antibody: ocrelizumab/ofatumumab/rituximab; ^b^—referring to 1568 patients with detailed data on the DMD used (FU1); CLAD/DMF/TER—cladribine/dimethyl fumarate/teriflunomide; ^c^—referring to 1360 patients with detailed data on the DMD used (FU2); DMD—disease-modifying drug; FU—follow-up; IFNβ/GLAT—interferon beta-1a/interferon beta-1b/peginterferon beta-1a/glatiramer acetate; MS—multiple sclerosis; N—number of patients; PDDS—patient-determined disease steps; PPMS—primary progressive MS; RRMS—relapsing–remitting MS; S1P RM—sphingosin-1-phosphate receptor modulator: fingolimod/ozanimod/siponimod; SARS-CoV-2—severe acute respiratory syndrome coronavirus 2; SPMS—secondary progressive MS; X^1^—first SARS-CoV-2 vaccination.

**Table 2:** Vaccination scheme and time between vaccine doses among 2212 MS patients with complete information on vaccination.

Vaccination Scheme	N (%)	Time between X^1^ and X^2^ [Weeks] (25%, 75% Quantiles), Median
Tozinameran (BNT162b2, Comirnaty^®^ [BioNTech/Pfizer]), 2 doses	1717 (77.6)	5.5 (4.2, 5.5)
Elasomeran (mRNA-1273, Spikevax^®^ [Moderna]), 2 doses	218 (9.9)	5.5 (5.2, 5.5)
AZD1222 (Vaxzevria^®^ [AstraZeneca]), 2 doses	71 (3.2)	9.7 (8.3, 11.0)
Ad26.COV2.S (COVID-19 Vaccine Janssen [Janssen/Johnson & Johnson]), 1 dose	22 (1.0)	n.a.
Heterologous, 2 doses	184 (8.3)	10.1 (8.5, 11.0)

N—number of patients; n.a.—not available; X^1^—first SARS-CoV-2 vaccination; X^2^—second SARS-CoV-2 vaccination.

**Table 3:** Comparison of MS patients with and without relapses after SARS-CoV-2 vaccinations regarding demographic and clinical characteristics.

	PwMS	
	With Relapse after Vaccination (N = 244 *)	Without Relapse after Vaccination (N = 2414)
Gender, N (%)		
Female	203 (83.5)	1853 (77.4)
Male	39 (16.0)	534 (22.3)
Divers	1 (0.4)	8 (0.3)
Age [years], median	41.9	45.6
MS disease course, N (%)		
RRMS	206 (84.4)	1781 (73.8)
SPMS	28 (11.5)	428 (17.7)
PPMS	0 (0.0)	99 (4.1)
Undefined	10 (4.1)	106 (4.4)
Disability level (PDDS), N (%)		
Mild (0–1)	133 (54.5)	1243 (51.5)
Moderate (2–4)	90 (36.9)	873 (36.2)
Severe (≥5)	21 (8.6)	298 (12.3)
Coincident autoimmune disease, N (%)	60 (24.6)	510 (21.1)
DMD treatment, N (%)	146 (60.1)	1774 (73.5)
Relapse within the year prior to X^1^, N (%)	69 (28.3)	322 (13.3)
Relapse within 6 months prior to X^1^, N (%)	43 (17.6)	170 (7.0)
Relapse within 3 months prior to X^1^, N (%)	18 (7.4)	82 (3.4)
Time from last relapse (before X^1^) to X^1^ [years], median (range)	1.4 (0.05–24.2)	3.3 (0.03–40.7)

DMD, disease-modifying drug; MS—multiple sclerosis; N—number of patients; PDDS—patient-determined disease steps; PPMS—primary progressive MS; PwMS—people with MS; RRMS—relapsing–remitting MS; SARS-CoV-2—severe acute respiratory syndrome coronavirus 2; SPMS—secondary progressive MS; X^1^—first SARS-CoV-2 vaccination; *—three patients were excluded from this analysis due to implausibility.

## Data Availability

Anonymized data will be made available on request by any qualified investigator under the terms of the registries’ usage and access guidelines and subject to informed consent of the patients.

## Supplementary Materials

The following supporting information can be downloaded at: https://www.mdpi.com/article/10.3390/jcm12113640/s1, Supplementary Document S1: Baseline questionnaire; Supplementary Document S2: First follow-up questionnaire; Supplementary Document S3: Second follow-up questionnaire; Supplementary Document S4: SARS-CoV-2 vaccine distribution over time; Supplementary Document S5: SARS-CoV-2 infections following vaccination; Supplementary Document S6: SARS-CoV-2 vaccination reactions; Supplementary Document S7: Generalizability of patient cohort composition; Supplementary Figure S1: Patient characteristics associated with the occurrence of relapses in a univariable logistic regression model; Supplementary Table S1: Loss of patients across follow-ups; Supplementary Table S2: Matched pre-pandemic reference registry cohort from 2020 (N = 2182); Supplementary Table S3: Reference registry cohort of vaccinated MS patients (N = 615).
