# Supplementary material for: Frequency and Predictors of Relapses following SARS-CoV-2 Vaccination in Patients with Multiple Sclerosis: Interim Results from a Longitudinal Observational Study
Source: J Clin Med. 2023 May 24;12(11):3640. doi: 10.3390/jcm12113640 (PMC10254005; doi:10.3390/jcm12113640)
Supplement: Supplementary file 1 [file jcm-12-03640-s001.zip › REV_Supp Doc S1.pdf]

Supplementary Document S1. Baseline questionnaire

Gender:	Male	Female	Divers
Birth date:	__.____ (MM.JJJJ)		
Please indicate the federal state in which you currently live:	Baden-Wuerttemberg<br>Berlin<br>Bremen<br>Hesse<br>Lower Saxony<br>Rhineland-Palatinate<br>Saxony<br>Schleswig-Holstein<br>Bavaria	Brandenburg<br>Hamburg<br>Mecklenburg-Western Pomerania<br>North Rhine-Westphalia<br>Saarland<br>Saxony-Anhalt<br>Thuringia	

MS diagnosis

<table>
<tr><td>Date of first symptoms:</td><td>__.____ (MM.JJJJ)</td></tr>
<tr><td>Date of diagnosis</td><td>__.____ (MM.JJJJ)</td></tr>
<tr><td rowspan="3">Please indicate your MS disease course:</td><td>

Relapsing-remitting
In the relapsing-remitting course, sudden impairments occur that can last for a few days or weeks (so-called relapses). There can be weeks or months between the individual relapses. Relapsing-remitting means that the impairments become weaker or can disappear completely.

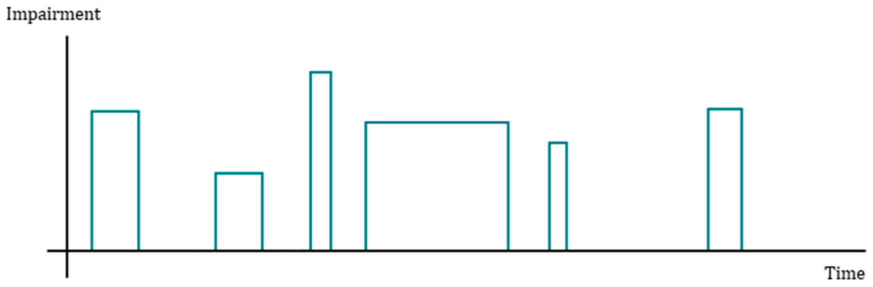

</td></tr>
<tr><td>

Secondary progressive
The secondary progressive course usually follows relapsing-remitting MS and is characterised by a decrease in the frequency of relapses (which may not occur at all), as well as a gradual worsening of disability.

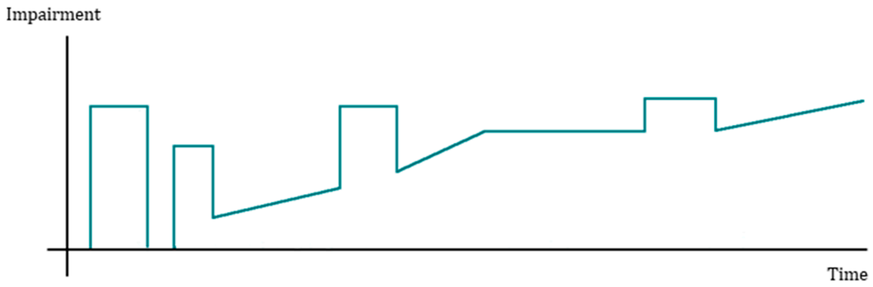

</td></tr>
<tr><td>

Primary progressive
In the primary chronic-progressive course, the impairment constantly increases.

</td></tr>
</table>

	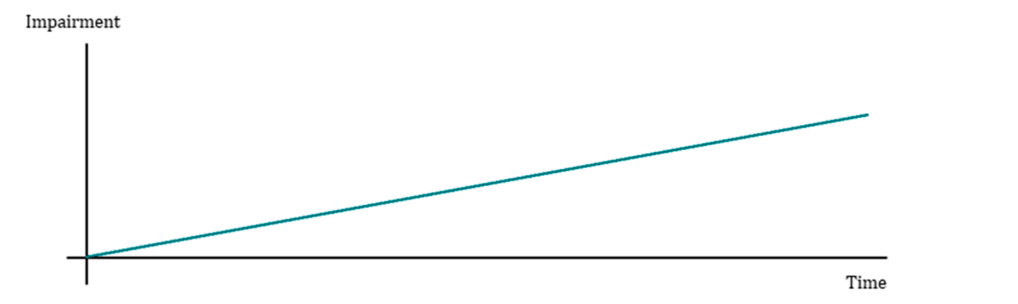
	**Undefined**

Patient-determined disease steps (PDDS)

Please read the choices below and choose the one that describes your own situation most appropriately.

This scale focuses mainly on how well you can **walk**.

You may not find a description that accurately reflects your condition, but please mark the category that describes your situation most closely.

Please choose one of the following answers:	
**0 Normal**<br>I may have some mild symptoms, mainly sensory due to my MS, but they do not limit my activity. When I have an episode, I return to normal as soon as the relapse is over.	
**1 Mild disability**<br>I have some noticeable symptoms due to my MS, but they are minor and have a small impact on my lifestyle.	
**2 Moderate disability**<br>I have no limitations in my ability to walk. However, I have significant problems due to MS that limit daily activities in other ways.	
**3 Walking impairment**<br>MS affects my activities, especially walking. I can work all day but sporting or physically demanding activities are more difficult than before. Normally I do not need a walking stick or other aids to walk, but I might need some help during a relapse.	
**4 Occasional use of a walking aid (walking stick use)**<br>I use a walking stick, a single crutch or some other form of support (e.g., touching a wall or leaning on someone's arm) to walk all or part of the time, especially when I walk outdoors. I think I can walk 8 metres in 20 seconds without a walking stick or crutch. I always need some help (walking stick or crutch) when I want to walk up to 300 metres.	
**5 Walking aid dependency**<br>To walk 8 metres, I need a walking stick, a crutch or someone to hold on to. I can move around the house or other buildings by holding onto furniture or touching the walls to support myself. I can use a scooter or wheelchair if I want to travel longer distances.	
**6 Bilateral support**<br>To walk up to 8 metres, I need two walking sticks or crutches or a rollator. For longer distances I can use a scooter or wheelchair.	
**7 Wheelchair**<br>My main form of mobility is a wheelchair. I may be able to stand and/or take a step or two, but I cannot walk 8 metres, even with crutches or a rollator.	
**8 Bedriddenness**<br>I cannot sit in a wheelchair for more than an hour.	

Immunomodulatory treatment

Are you being or have you been treated with immunotherapeutics?			
Yes, currently	Yes, in the past	Yes, currently and in the past	No, never

Current immunotherapy	
When did you start treatment?	__.____ (MM.JJJJ)
**Active substance / preparation:**	
Alemtuzumab / Lemtrada	Azathioprine / Imurek
Cladribine / Mavenclad	Cyclophosphamide / Endoxan
Daclizumab / Zinbryta	Dimethyl fumarate / Tecfidera
Fingolimod / Gilenya	Glatiramer acetate / Clift
Glatiramer acetate / Copaxone	Immunoglobulin / IVIG / Octagam
Interferon beta-1a / Avonex	Interferon beta-1a / Rebif22
Interferon beta-1a / Rebif44	Interferon beta-1b / Betaferon
Interferon beta-1b / Extavia	Methotrexate
Mitoxantrone / Novatron	Mitoxantrone / Ralenova
Natalizumab / Tysabri	Ocrelizumab / Ocrevus
Ofatumumab / Kesimpta	Ozanimod / Zeposia
Peginterferon beta-1a / Plegridy	Rituximab / MabThera
Siponimod / Mayzent	Steroids - Long-term therapy, longer than 2 months
Steroids – Pulse therapy	Steroids intrathecal
Teriflunomide / Aubagio	Other: ____________________

Immunotherapy in the past	
Until when were you treated?	__.____ (MM.JJJJ)
**Active substance / preparation:**	
Alemtuzumab / Lemtrada	Azathioprine / Imurek
Cladribine / Mavenclad	Cyclophosphamide / Endoxan
Daclizumab / Zinbryta	Dimethyl fumarate / Tecfidera
Fingolimod / Gilenya	Glatiramer acetate / Clift
Glatiramer acetate / Copaxone	Immunoglobulin / IVIG / Octagam
Interferon beta-1a / Avonex	Interferon beta-1a / Rebif22
Interferon beta-1a / Rebif44	Interferon beta-1b / Betaferon
Interferon beta-1b / Extavia	Methotrexate
Mitoxantrone / Novatron	Mitoxantrone / Ralenova
Natalizumab / Tysabri	Ocrelizumab / Ocrevus
Ofatumumab / Kesimpta	Ozanimod / Zeposia
Peginterferon beta-1a / Plegridy	Rituximab / MabThera
Siponimod / Mayzent	Steroids - Long-term therapy, longer than 2 months
Steroids – Pulse therapy	Steroids intrathecal
Teriflunomide / Aubagio	Other: ____________________

Comorbidities

Do you suffer from allergies?	Yes	No
If yes, what allergies do you suffer from?		
Drug allergy	House dust / pollen	

Food	Nickel / fragrances
Insect bites	
Are there other autoimmune diseases?	
☐ Thyroid diseases (Hashimoto's thyroiditis, Graves' disease)	☐ Psoriasis
☐ Chronic polyarthritis (rheumatism)	☐ Inflammatory bowel disease (Ulcerative colitis, Crohn's disease)
☐ None	☐ Other: ______________________

Relapses prior to the SARS-CoV-2 vaccination

Last MS relapse prior to the vaccination:<br>If you do not know the date,<br>please do not provide any information here.	__.____ (MM.JJJJ)
A relapse is characterised by the appearance of new symptoms or the worsening of existing symptoms that last longer than 24 hours. These symptoms can vary greatly and usually develop within a few hours or days. After the relapse, the symptoms may decrease or disappear completely, depending on the course of the disease. To be able to assume that there is another relapse, there must be at least 30 consecutive days between relapses.	

SARS-CoV-2 infections prior to the vaccination

Have you been diagnosed with a SARS-CoV-2 infection before the first vaccination?	
Yes, via a PCR test (nasal/pharyngeal swab with laboratory examination)	Yes, via an antigen test (rapid test)
Yes, via an antibody test (blood sample and laboratory examination)	Yes, without a confirmed test, but symptoms
No	
Usually, patients who have already been infected with SARS-CoV-2 are vaccinated only once.	

First SARS-CoV-2 vaccine administered

Comirnaty (BioNTech/Pfizer)	Spikevax (Moderna)
Vaxzevria (AstraZeneca)	COVID-19 Vaccine Janssen (Janssen-Cilag International)
Comirnaty (BioNTech/Pfizer) and flu vaccination	Spikevax (Moderna) and flu vaccination
Vaxzevria (AstraZeneca) and flu vaccination	COVID-19 Vaccine Janssen (Janssen-Cilag International) and flu vaccination

First vaccination date

Date:	__.__.____ (TT.MM.JJJJ)
**Where were you vaccinated?**	
Vaccination centre	Residential/nursing home (as resident)
Medical facility (as an employee)	Medical facility (as a patient)
General practitioner or specialist	
Has it been confirmed by the vaccination centre / vaccinating institution that you have already survived a SARS-CoV-2 infection and therefore only need one dose?	
Yes	No

Vaccination reactions

**Did you experience any vaccination reactions in connection with the first vaccination?**	
Yes	No
Please select the information below that applies to you.	
☐ **Emesis / vomitus**, occurred after _____ days	
o Subsided after ________ days	o Still persistent
☐ **Rash / exanthema**, occurred after _____ days	
o Subsided after ________ days	o Still persistent
☐ **Swollen lymph nodes / lymphadenopathy**, occurred after _____ days	
o Subsided after ________ days	o Still persistent
☐ **Palpitation / tachycardia**, occurred after _____ days	
o Subsided after ________ days	o Still persistent
☐ **Local reaction**, occurred after _____ days	
o Subsided after ________ days	o Still persistent
☐ **Malaise**, occurred after _____ days	
o Subsided after ________ days	o Still persistent
☐ **Melalgia / limb pain**, occurred after _____ days	
o Subsided after ________ days	o Still persistent
☐ **Nausea**, occurred after _____ days	
o Subsided after ________ days	o Still persistent
☐ **Asthenia**, occurred after _____ days	
o Subsided after ________ days	o Still persistent
☐ **Vertigo**, occurred after _____ days	
o Subsided after ________ days	o Still persistent
☐ **Pain at the injection area**, occurred after _____ days	
o Subsided after ________ days	o Still persistent
☐ **Muscular pain / myalgia**, occurred after _____ days	
o Subsided after ________ days	o Still persistent
☐ **Flu-like symptoms**, occurred after _____ days	
o Subsided after ________ days	o Still persistent
☐ **Fatigue**, occurred after _____ days	
o Subsided after ________ days	o Still persistent
☐ **Ague / undulant fever**, occurred after _____ days	
o Subsided after ________ days	o Still persistent
☐ **Headache**, occurred after _____ days	
o Subsided after ________ days	o Still persistent
☐ **Fever / pyrexia**, occurred after _____ days	
o Subsided after ________ days	o Still persistent
☐ **Other**, occurred after _____ days	
o Subsided after ________ days	o Still persistent

New-onset MS symptoms

Did you experience any new MS symptoms in connection with the first vaccination?	
Yes	No
Please select the information below that applies to you.	
**Impaired walking impairment**, occurred after _____ days	Still persistent
Subsided after ________ days	
**Spasticity**, occurred after _____ days	Still persistent
Subsided after ________ days	
**Movement disorders / ataxia / tremor**, occurred after _____ days	Still persistent
Subsided after ________ days	

**Fatigue**, occurred after _____ days	Still persistent
Subsided after ________ days	
**Pain**, occurred after _____ days	Still persistent
Subsided after ________ days	
**Bladder dysfunction / impaired micturition**, occurred after _____ days	Still persistent
Subsided after ________ days	
**Bowel dysfunction / impaired defecation**, occurred after _____ days	Still persistent
Subsided after ________ days	
**Sexual disorders**, occurred after _____ days	Still persistent
Subsided after ________ days	
**Cognitive disorders**, occurred after _____ days	Still persistent
Subsided after ________ days	
**Depression**, occurred after _____ days	Still persistent
Subsided after ________ days	
**Eye movement disorders / oculomotor disorders**, occurred after _____ days	Still persistent
Subsided after ________ days	
**Speaking and voice disorders/ dysarthria / dysphonia**, occurred after _____ days	Still persistent
Subsided after ________ days	
**Swallowing disorders / dysphagia**, occurred after _____ days	Still persistent
Subsided after ________ days	
**Epileptic seizures**, occurred after _____ days	Still persistent
Subsided after ________ days	
**Palsy / paresis**, occurred after _____ days	Still persistent
Subsided after ________ days	
**Optic nerve inflammation / optic neuritis**, occurred after _____ days	Still persistent
Subsided after ________ days	
**Other seizure-like symptoms / paroxysms**, occurred after _____ days	Still persistent
Subsided after ________ days	

Worsened MS symptoms

Have you experienced any worsening of MS symptoms in relation to the first vaccination?	
Yes	No
Please select the information below that applies to you.	
**Impaired walking impairment**, occurred after _____ days	Still persistent
Subsided after ________ days	
**Spasticity**, occurred after _____ days	Still persistent
Subsided after ________ days	
**Movement disorders / ataxia / tremor**, occurred after _____ days	Still persistent
Subsided after ________ days	
**Fatigue**, occurred after _____ days	Still persistent

Subsided after ________ days	
**Pain**, occurred after _____ days	Still persistent
Subsided after ________ days	
**Bladder dysfunction / impaired micturition**, occurred after _____ days	Still persistent
Subsided after ________ days	
**Bowel dysfunction / impaired defecation**, occurred after _____ days	Still persistent
Subsided after ________ days	
**Sexual disorders**, occurred after _____ days	Still persistent
Subsided after ________ days	
**Cognitive disorders**, occurred after _____ days	Still persistent
Subsided after ________ days	
**Depression**, occurred after _____ days	Still persistent
Subsided after ________ days	
**Eye movement disorders / oculomotor disorders**, occurred after _____ days	Still persistent
Subsided after ________ days	
**Speaking and voice disorders/ dysarthria / dysphonia**, occurred after _____ days	Still persistent
Subsided after ________ days	
**Swallowing disorders / dysphagia**, occurred after _____ days	Still persistent
Subsided after ________ days	
**Epileptic seizures**, occurred after _____ days	Still persistent
Subsided after ________ days	
**Palsy / paresis**, occurred after _____ days	Still persistent
Subsided after ________ days	
**Optic nerve inflammation / optic neuritis**, occurred after _____ days	Still persistent
Subsided after ________ days	
**Other seizure-like symptoms / paroxysms**, occurred after _____ days	Still persistent
Subsided after ________ days	

Relapses following the first vaccination

Did you experience MS attacks after the first vaccination?	Yes	No
A relapse is characterised by the appearance of new symptoms or the worsening of existing symptoms that last longer than 24 hours. These symptoms can vary greatly and usually develop within a few hours or days. After the relapse, the symptoms may decrease or disappear completely, depending on the course of the disease. To be able to assume that there is another relapse, there must be at least 30 consecutive days between relapses.		
If yes:		
How many relapses occurred?	____________	
Have you received a steroid therapy in connection with these MS relapses?	Yes	No

SARS-CoV-2 infections following the first vaccination

Did you have a confirmed SARS-CoV-2 infection after the first vaccination?	Yes	No
If yes:		
Which test was conducted?		
PCR test	Antigen Test	
Antibody test	Unknown	
Please indicate the onset of SARS-CoV-2 infection:	__.____ (MM.JJJJ)	
What symptoms did you experience?		
Increased temperature	Cough	
Lassitude	Joint, bone or muscle pain	
Headache	Sore throat	
Shortness of breath	Blocked nose	
Chills	Loss of smell or taste	
Pneumonia	Other: ______________________	
For the treatment you were:		
Admitted as an inpatient in a hospital		
Admitted to hospital as an inpatient and treated in intensive care for at least one day.		
Treated at home.		
If they have been admitted to a hospital:		
How many days were you admitted to hospital?	________________	
Were you connected to a (ventilated) respirator or did you receive oxygen therapy during your inpatient stay?		
Non-invasive treatment	Invasive treatment	
Non-invasive ventilation: respiratory support or ventilation without invasive ventilation access, for example via a mask placed over the mouth and nose. Invasive ventilation: Placement of a breathing tube inserted through the mouth or nose to serve as an artificial airway.		
How many days were you ventilated?	___________________	
Do you still suffer from concomitant symptoms after your SARS-CoV-2 infection?	Yes	No
If so, which symptoms do you still suffer from?		
Increased temperature	Cough	
Lassitude	Joint, bone or muscle pain	
Headache	Sore throat	
Shortness of breath	Blocked nose	
Chills	Loss of smell or taste	
Pneumonia	Other: ______________________	
