# Supplementary material for: Frequency and Predictors of Relapses following SARS-CoV-2 Vaccination in Patients with Multiple Sclerosis: Interim Results from a Longitudinal Observational Study
Source: J Clin Med. 2023 May 24;12(11):3640. doi: 10.3390/jcm12113640 (PMC10254005; doi:10.3390/jcm12113640)
Supplement: Supplementary file 1 [file jcm-12-03640-s001.zip › REV_Supp Doc S3.pdf]

Supplementary Document S3. Second follow-up questionnaire

SARS-CoV-2 infections following the SARS-CoV-2 vaccination

For vaccines that require two vaccinations:			
Did you have a confirmed SARS-CoV-2 infection following the vaccination?	Yes, following the first vaccination	Yes, following the second vaccination	No
For vaccines that require one vaccination:			
Did you have a confirmed SARS-CoV-2 infection following the vaccination?	Yes	No	

If yes:		
Which test was conducted?		
PCR test	Antigen Test	
Antibody test	Unknown	
Please indicate the onset of SARS-CoV-2 infection:		__.____ (MM.JJJJ)
What symptoms did you experience?		
Increased temperature	Cough	
Lassitude	Joint, bone or muscle pain	
Headache	Sore throat	
Shortness of breath	Blocked nose	
Chills	Loss of smell or taste	
Pneumonia	Other: ____________________	
For the treatment you were:		
Admitted as an inpatient in a hospital		
Admitted to hospital as an inpatient and treated in intensive care for at least one day.		
Treated at home.		
If they have been admitted to a hospital:		
How many days were you admitted to hospital?	_______________	
Were you connected to a (ventilated) respirator or did you receive oxygen therapy during your inpatient stay?		
Non-invasive treatment	Invasive treatment	No ventilation
Non-invasive ventilation: respiratory support or ventilation without invasive ventilation access, for example via a mask placed over the mouth and nose.<br>Invasive ventilation: Placement of a breathing tube inserted through the mouth or nose to serve as an artificial airway.		
How many days were you ventilated?	__________________	
Do you still suffer from concomitant symptoms after your SARS-CoV-2 infection?	Yes	No
If so, which symptoms do you still suffer from?		
Increased temperature	Cough	
Lassitude	Joint, bone or muscle pain	
Headache	Sore throat	
Shortness of breath	Blocked nose	
Chills	Loss of smell or taste	
Pneumonia	Other: ____________________	

Antibody test following the vaccination

Did you have an antibody test after a vaccination?		
Yes, antibodies positive	Yes, antibodies negative	No
If yes:		
Date of your (last) antibody test:	__.__.____ (TT/MM/JJJJ)	

New-onset MS symptoms

Did you experience any new MS symptoms in connection with the last vaccination?	
Yes	No
Please select the information below that applies to you.	
**Impaired walking impairment**, occurred after _____ days	Still persistent
Subsided after ________ days	
**Spasticity**, occurred after _____ days	Still persistent
Subsided after ________ days	
**Movement disorders / ataxia / tremor**, occurred after _____ days	Still persistent
Subsided after ________ days	
**Fatigue**, occurred after _____ days	Still persistent
Subsided after ________ days	
**Pain**, occurred after _____ days	Still persistent
Subsided after ________ days	
**Bladder dysfunction / impaired micturition**, occurred after _____ days	Still persistent
Subsided after ________ days	
**Bowel dysfunction / impaired defecation**, occurred after _____ days	Still persistent
Subsided after ________ days	
**Sexual disorders**, occurred after _____ days	Still persistent
Subsided after ________ days	
**Cognitive disorders**, occurred after _____ days	Still persistent
Subsided after ________ days	
**Depression**, occurred after _____ days	Still persistent
Subsided after ________ days	
**Eye movement disorders / oculomotor disorders**, occurred after _____ days	Still persistent
Subsided after ________ days	
**Speaking and voice disorders/ dysarthria / dysphonia**, occurred after _____ days	Still persistent
Subsided after ________ days	
**Swallowing disorders / dysphagia**, occurred after _____ days	Still persistent
Subsided after ________ days	
**Epileptic seizures**, occurred after _____ days	Still persistent
Subsided after ________ days	
**Palsy / paresis**, occurred after _____ days	Still persistent
Subsided after ________ days	
**Optic nerve inflammation / optic neuritis**, occurred after _____ days	Still persistent
Subsided after ________ days	
**Other seizure-like symptoms / paroxysms**, occurred after _____ days	Still persistent
Subsided after ________ days	

Worsened MS symptoms

Have you experienced any worsening of MS symptoms in relation to the last vaccination?	
Yes	No
Please select the information below that applies to you.	
**Impaired walking impairment**, occurred after _____ days	Still persistent
Subsided after ________ days	
**Spasticity**, occurred after _____ days	Still persistent
Subsided after ________ days	
**Movement disorders / ataxia / tremor**, occurred after _____ days	Still persistent
Subsided after ________ days	
**Fatigue**, occurred after _____ days	Still persistent
Subsided after ________ days	
**Pain**, occurred after _____ days	Still persistent
Subsided after ________ days	
**Bladder dysfunction / impaired micturition**, occurred after _____ days	Still persistent
Subsided after ________ days	
**Bowel dysfunction / impaired defecation**, occurred after _____ days	Still persistent
Subsided after ________ days	
**Sexual disorders**, occurred after _____ days	Still persistent
Subsided after ________ days	
**Cognitive disorders**, occurred after _____ days	Still persistent
Subsided after ________ days	
**Depression**, occurred after _____ days	Still persistent
Subsided after ________ days	
**Eye movement disorders / oculomotor disorders**, occurred after _____ days	Still persistent
Subsided after ________ days	
**Speaking and voice disorders/ dysarthria / dysphonia**, occurred after _____ days	Still persistent
Subsided after ________ days	
**Swallowing disorders / dysphagia**, occurred after _____ days	Still persistent
Subsided after ________ days	
**Epileptic seizures**, occurred after _____ days	Still persistent
Subsided after ________ days	
**Palsy / paresis**, occurred after _____ days	Still persistent
Subsided after ________ days	
**Optic nerve inflammation / optic neuritis**, occurred after _____ days	Still persistent
Subsided after ________ days	
**Other seizure-like symptoms / paroxysms**, occurred after _____ days	Still persistent
Subsided after ________ days	

Relapses following the last vaccination

Did you experience MS attacks after the last vaccination?	Yes	No
A relapse is characterised by the appearance of new symptoms or the worsening of existing symptoms that last longer than 24 hours. These symptoms can vary greatly and usually develop within a few hours or days. After the relapse, the symptoms may decrease or disappear completely, depending on the course of the disease. To be able to assume that there is another relapse, there must be at least 30 consecutive days between relapses.		
If yes:		
How many relapses occurred?	____________	
What MS symptoms have you experienced during the relapses?<br>Please select the information below that applies to you.		
Symptoms experienced:		
Epileptic seizures		
Bowel dysfunction/impaired defecation		
Sexual disorders		
Swallowing disorders/dysphagia		
Speaking and voice disorders/dysarthria/dysphonia		
Depression		
Optic nerve inflammation/optic neuritis		
Bladder dysfunction/impaired micturition		
Eye movement disorders/oculomotor disorders		
Palsy/paresis		
Other seizure-like symptoms/paroxysms		
Movement disorders/ataxia/tremor		
Cognitive disorders		
Spasticity		
Pain		
Impaired walking impairment		
Fatigue		
Have you received a steroid therapy in connection with these MS relapses?	Yes	No
Was the relapse diagnosed by a doctor?	Yes	No

Patient-determined disease steps (PDDS)

Please read the choices below and choose the one that describes your own situation most appropriately.

This scale focuses mainly on how well you can **walk**.

You may not find a description that accurately reflects your condition, but please mark the category that describes your situation most closely.

Please choose one of the following answers:	
0 **Normal**<br>I may have some mild symptoms, mainly sensory due to my MS, but they do not limit my activity. When I have an episode, I return to normal as soon as the relapse is over.	
1 **Mild disability**<br>I have some noticeable symptoms due to my MS, but they are minor and have a small impact on my lifestyle.	
2 **Moderate disability**<br>I have no limitations in my ability to walk. However, I have significant problems due to MS that limit daily activities in other ways.	

3 **Walking impairment**<br>MS affects my activities, especially walking. I can work all day but sporting or physically demanding activities are more difficult than before. Normally I do not need a walking stick or other aids to walk, but I might need some help during a relapse.	
4 **Occasional use of a walking aid (walking stick use)**<br>I use a walking stick, a single crutch or some other form of support (e.g., touching a wall or leaning on someone's arm) to walk all or part of the time, especially when I walk outdoors. I think I can walk 8 metres in 20 seconds without a walking stick or crutch. I always need some help (walking stick or crutch) when I want to walk up to 300 metres.	
5 **Walking aid dependency**<br>To walk 8 metres, I need a walking stick, a crutch or someone to hold on to. I can move around the house or other buildings by holding onto furniture or touching the walls to support myself. I can use a scooter or wheelchair if I want to travel longer distances.	
6 **Bilateral support**<br>To walk up to 8 metres, I need two walking sticks or crutches or a rollator. For longer distances I can use a scooter or wheelchair.	
7 **Wheelchair**<br>My main form of mobility is a wheelchair. I may be able to stand and/or take a step or two, but I cannot walk 8 metres, even with crutches or a rollator.	
8 **Bedriddenness**<br>I cannot sit in a wheelchair for more than an hour.	
