# Supplementary material for: Frequency and Predictors of Relapses following SARS-CoV-2 Vaccination in Patients with Multiple Sclerosis: Interim Results from a Longitudinal Observational Study
Source: J Clin Med. 2023 May 24;12(11):3640. doi: 10.3390/jcm12113640 (PMC10254005; doi:10.3390/jcm12113640)
Supplement: Supplementary file 1 [file jcm-12-03640-s001.zip › REV_Supp Doc S5.pdf]

Supplementary Document S5. SARS-CoV-2 infections following vaccination

Eight people with multiple sclerosis (PwMS) reported a SARS-CoV-2 infection following vaccination over a median follow-up time of four months: six after the first vaccination and two after the second vaccination. The infections were detected by polymerase chain reaction test in six patients and antibody test in one patient. The remaining patient provided no data on the test type. Five of the eight patients were treated with disease-modifying drugs (DMDs) at the time of the infection (glatiramer acetate: two patients; ocrelizumab, fingolimod, siponimod: one patient each), and three were without an immunomodulatory treatment. SARS-CoV-2 infections occurred within two months after the first vaccination in five patients while the remaining three PwMS did not provide data on infection dates. All SARS-CoV-2 infections reported until the data export date occurred from February to June 2021.

The lower rate of breakthrough infections (N=2, 0.1%) in our study after a fully administered SARS-CoV-2 vaccination compared with findings from the general population (Hacisuleyman et al.: 0.5%, Geysels et al.: 0.3%) [1,2] may be explained by (i) the short interval between complete immunization and the subsequent observation and reporting time captured in the present study with potentially still-well-preserved immunity against SARS-CoV2 [3,4], (ii) the observation period in our study (May to October) that lies outside the seasonal peak of SARS-CoV-2 infections in Germany from November to April [5] and (iii) the fact that only clinically manifest vaccination breakthroughs were documented in our study. In the continuation of our study, we expect more robust longitudinal results on these aspects for clinical practice [6]. Further differences in the frequencies of reported vaccination reactions (Supplementary Document S6) compared with the previous studies by Lotan et al. [7] and Achiron et al. [8] may be due to differences in data access (country-wide online survey vs. single specialized clinic vs. country-wide healthcare database) and data collection types (patient-reported vs. physician/neurologist-reported).

<u>References</u>

1. Hacisuleyman, E.; Hale, C.; Saito, Y.; Blachere, N.E.; Bergh, M.; Conlon, E.G.; Schaefer-Babajew, D.J.; DaSilva, J.; Muecksch, F.; Gaebler, C.; et al. Vaccine Breakthrough Infections with SARS-CoV-2 Variants. *N Engl J Med* **2021**, *384*, 2212–2218, doi:10.1056/NEJMoa2105000.

2. Geysels, D.; Van Damme, P.; Verstrepen, W.; Bruynseels, P.; Janssens, B.; Smits, P.; Naesens, R. SARS-CoV-2 Vaccine Breakthrough Infections among Healthcare Workers in a Large Belgian Hospital Network. *Infect Control Hosp Epidemiol* **2021**, *43*, 1755–1757, doi:10.1017/ice.2021.326.
3. Nordström, P.; Ballin, M.; Nordström, A. Effectiveness of Heterologous ChAdOx1 NCoV-19 and MRNA Prime-Boost Vaccination against Symptomatic Covid-19 Infection in Sweden: A Nationwide Cohort Study. *Lancet Reg Health Eur* **2021**, *11*, 100249, doi:10.1016/j.lanepe.2021.100249.
4. Collier, D.A.; Ferreira, I.A.T.M.; Kotagiri, P.; Datir, R.P.; Lim, E.Y.; Touizer, E.; Meng, B.; Abdullahi, A.; CITIID-NIHR BioResource COVID-19 Collaboration; Elmer, A.; et al. Age-Related Immune Response Heterogeneity to SARS-CoV-2 Vaccine BNT162b2. *Nature* **2021**, *596*, 417–422, doi:10.1038/s41586-021-03739-1.
5. Robert Koch Institute. COVID-19-Dashboard. Available online: https://experience.arcgis.com/experience/478220a4c454480e823b17327b2bf1d4 (accessed on 12 April 2023).
6. Couzin-Frankel, J. Will COVID-19 Change Science? Past Pandemics Offer Clues. *Science* **2021**, *373*, 264–265, doi:10.1126/science.373.6552.264.
7. Lotan, I.; Wilf-Yarkoni, A.; Friedman, Y.; Stiebel-Kalish, H.; Steiner, I.; Hellmann, M.A. Safety of the BNT162b2 COVID-19 Vaccine in Multiple Sclerosis (MS): Early Experience from a Tertiary MS Center in Israel. *Eur J Neurol* **2021**, *28*, 3742–3748, doi:10.1111/ene.15028.
8. Achiron, A.; Dolev, M.; Menascu, S.; Zohar, D.-N.; Dreyer-Alster, S.; Miron, S.; Shirbint, E.; Magalashvili, D.; Flechter, S.; Givon, U.; et al. COVID-19 Vaccination in Patients with Multiple Sclerosis: What We Have Learnt by February 2021. *Mult Scler* **2021**, *27*, 864–870, doi:10.1177/13524585211003476.
