# Supplementary material for: Frequency and Predictors of Relapses following SARS-CoV-2 Vaccination in Patients with Multiple Sclerosis: Interim Results from a Longitudinal Observational Study
Source: J Clin Med. 2023 May 24;12(11):3640. doi: 10.3390/jcm12113640 (PMC10254005; doi:10.3390/jcm12113640)
Supplement: Supplementary file 1 [file jcm-12-03640-s001.zip › REV_Supp Doc S6.pdf]

Supplementary Document S6. SARS-CoV-2 vaccination reactions

The frequencies of specific vaccination reactions were analysed proportionately for the baseline survey (after the first vaccination) as well as the first follow-up (after the second vaccination) and compared using the McNemar test. If the end dates of the vaccination reactions were not reported by the participants, then those participants were censored at the completion date of the respective survey. P-values were adjusted according to the false discovery rate [1] of 5% to correct for alpha error accumulation in multiple testing.

Generally, people with multiple sclerosis (PwMS) reported vaccination reactions (at least one) more often after the second (64.9%) than after the first SARS-CoV-2 vaccination (60.9%). This also applied to reporting at least five vaccination reactions (first vaccination: 20.0%, second: 28.5%). In a previous study, which assessed United Kingdom (UK) and German patients, UK patients reported more vaccination reactions after the first vaccination compared to the second one (48.7% vs. 30.0%) [2]. This may be due to the fact that UK patients more often received the vector-based vaccine AZD1222, whereas the vast majority of German patients received the mRNA-based vaccine tozinameran. In the present study, the most frequent vaccination reactions after both vaccinations were pain at the injection area (first: 46.4%, second: 48.7%) and fatigue (first: 33.3%, second: 39.7%) (Figure SD3). Fatigue, headache, asthenia, melalgia/limb pain, local reaction, malaise, muscular pain/myalgia, flu-like symptoms, vertigo, ague/undulant fever, fever/pyrexia, nausea, swollen lymph nodes/lymphadenopathy, palpitation/tachycardia and emesis/vomitus occurred notably more often after the second vaccination (McNemar test: $p \leq 0.038$). The p-values remained significant after adjustment according to the false discovery rate ($q \leq 0.046$). After the first SARS-CoV-2 vaccination, patients who received elasomeran (69.7%, Fisher's exact test: $p=0.005$) or AZD1222 (74.3%, $p<0.001$) reported vaccination reactions more often than patients who received tozinameran (60.5%). After the second vaccination, there were more reports of vaccination reactions in the following patient groups: elasomeran vs. tozinameran (86.5% vs. 63.2%, $p<0.001$), elasomeran vs. AZD1222 (86.5% vs. 42.5%, $p<0.001$) and tozinameran vs. AZD1222 (63.2% vs. 42.5%, $p<0.001$). The frequencies of specific vaccination reactions stratified by vaccine type are shown in Figure SD4. All vaccination reactions started within a median of one day after vaccination and persisted for a median of less than one to four days (Figure SD5). Patients vaccinated twice with tozinameran in our study reported pain at the injection area (61.3% vs. 46.4%) and fatigue (51.1% vs. 38.1%)more often, and less often myalgia (23.5% vs.

36.8%) as well as ague (14.3% vs. 29.3%) compared with data by Lotan et al. (chi-square test: p<0.001, respectively) [3]. Compared with the data by Achiron et al., pain at the injection area (first vaccination: 16.0% vs. 46.9%; second vaccination: 14.3% vs. 46.8%; chi-square test: p<0.001, respectively), fatigue (first: 9.2% vs. 31.0%; second: 15.9% vs. 38.6%; p<0.001, respectively), headache (first: 4.5% vs. 19.2%; second: 7.4% vs. 25.5%; p<0.001, respectively), melalgia/limb pain (first: 2.3% vs. 13.5%; second: 9.2% vs. 24.6%; p<0.001, respectively) and flu-like symptoms (first: 2.0% vs. 6.1%, p<0.001; second: 12.0% vs. 13.2%, p=0.534) occurred more frequently in the participants of our study who were vaccinated twice with tozinameran [4]. Drawing a comparison to UK patients, fatigue (first: 27.9%, second: 3.1%), headache (first: 26.5%, second: 1.9%), and pain (muscle/joint/other; first: 26.0%, second: 2.2%) were also the most common vaccination reactions in this cohort, but occurring more often after the first vaccination [2], as addressed above.

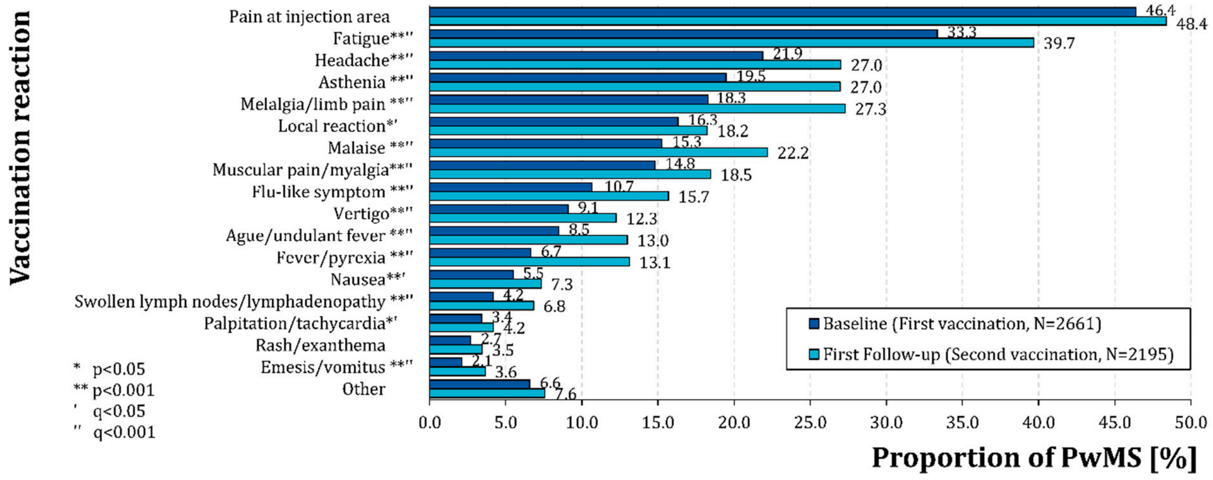

Figure SD3. Frequency of vaccination reactions after SARS-CoV-2 vaccination. Pain at injection area (46.4% vs. 48.4%) and fatigue (33.3% vs. 39.7%) were reported as the most frequent vaccination reactions after the first (dark blue) and second SARS-CoV-2 vaccinations (light blue). Differences in the frequency of vaccination reactions between the first and second vaccinations (calculated using the McNemar test) remained significant after p-value adjustment according to FDR (q≤0.046). FDR – false discovery rate; MS – multiple sclerosis; p – p-value, referring to patients providing complete data on vaccination reactions after both the first and second vaccinations; PwMS – people with MS; q – FDR-adjusted p-value; SARS-CoV-2 – severe acute respiratory syndrome coronavirus 2.

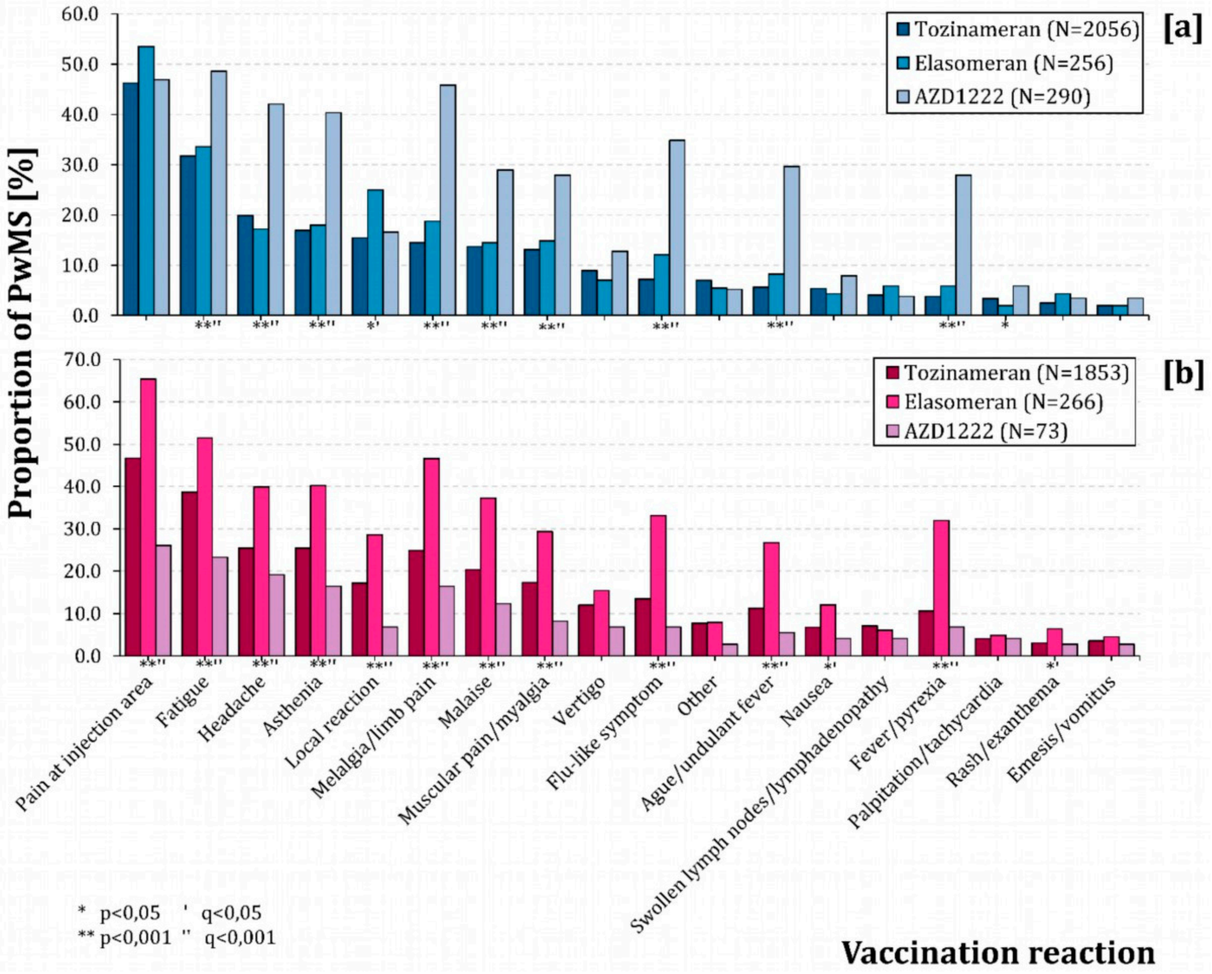

Figure SD4. Frequency of vaccination reactions after [a] the first and [b] second administration of SARS-CoV-2 vaccines analysed. Generally, the vaccination reactions after the first vaccination occurred more often in PwMS vaccinated with AZD1222 (vector vaccine) than in those vaccinated with tozinameran/BNT162b2 and elasomeran/mRNA-1273 (mRNA vaccines). After the second vaccination, PwMS vaccinated with elasomeran most frequently reported vaccination reactions. Differences in the frequency of vaccination reactions between the vaccine types were calculated using the chi-square test. The most frequent vaccination reactions after the first vaccination were pain at the injection area (tozinameran: 46.3%, elasomeran: 53.5%) as well as fatigue (AZD1222: 48.6%); after the second vaccination, it was also pain at the injection area (tozinameran: 46.8%, elasomeran: 65.4%, AZD1222: 26.0%). FDR – false discovery rate; p – p value; PwMS – patients with multiple sclerosis; q – FDR-adjusted p-value; SARS-CoV-2 – severe acute respiratory syndrome coronavirus 2.

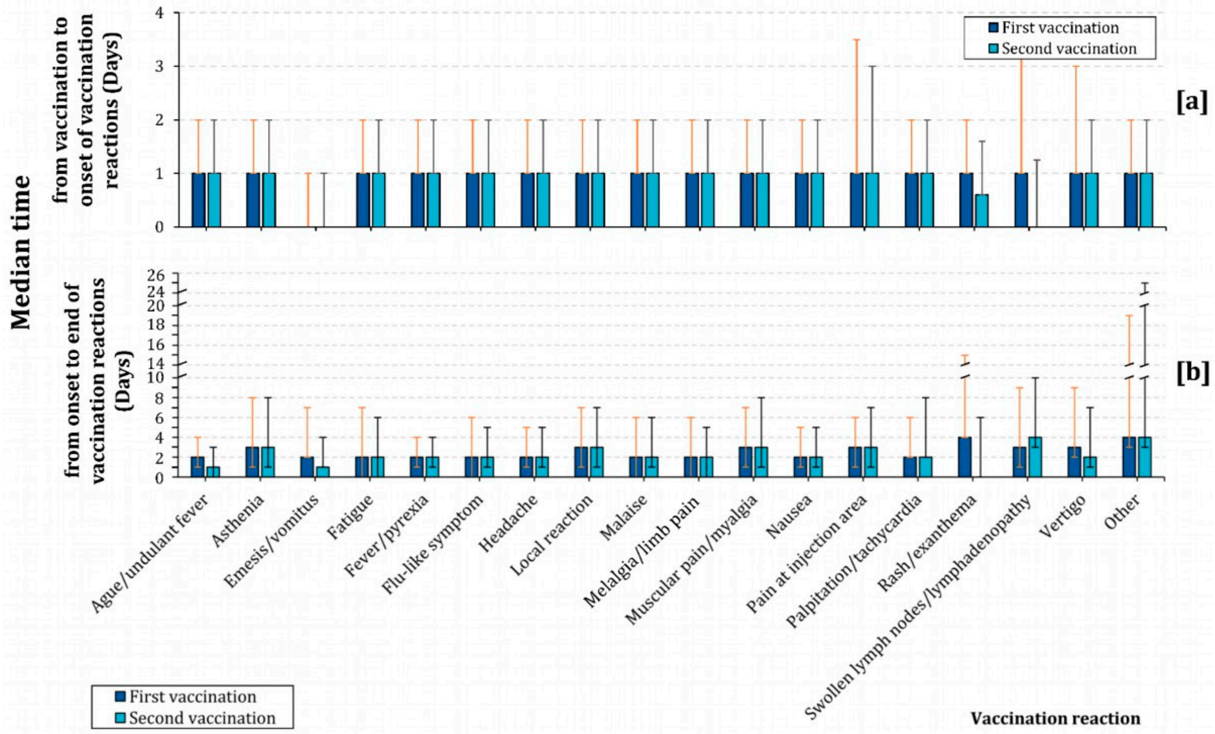

Figure SD5. Onset and duration of vaccination reactions. The bars show [a] the median time from the vaccination to the onset of the vaccination reactions as well as [b] the median time from the onset to the end of the vaccination reactions/the survey date (in case of symptoms still persisting at the date of participating into the survey, using the Kaplan-Meier method). The quantiles (25%, 75%) are symbolized by the whiskers (lower, upper). SARS-CoV-2 – severe acute respiratory syndrome coronavirus 2.

References

1. Benjamini, Y.; Hochberg, Y. Controlling the False Discovery Rate: A Practical and Powerful Approach to Multiple Testing. *J R Stat Soc Ser B Methodol* **1995**, *57*, 289–300.
2. Frahm, N.; Fneish, F.; Ellenberger, D.; Haas, J.; Loebermann, M.; Parciak, T.; Peters, M.; Pöhlau, D.; Rodgers, J.; Röper, A.-L.; et al. SARS-CoV-2 Vaccination in Patients with Multiple Sclerosis in Germany and the United Kingdom: Gender-Specific Results from a Longitudinal Observational Study. *Lancet Reg Health - Eur* **2022**, *22*, 100502, doi:10.1016/j.lanepe.2022.100502.
3. Lotan, I.; Wilf-Yarkoni, A.; Friedman, Y.; Stiebel-Kalish, H.; Steiner, I.; Hellmann, M.A. Safety of the BNT162b2 COVID-19 Vaccine in Multiple Sclerosis (MS): Early Experience from a Tertiary MS Center in Israel. *Eur J Neurol* **2021**, *28*, 3742–3748, doi:10.1111/ene.15028.
4. Achiron, A.; Dolev, M.; Menascu, S.; Zohar, D.-N.; Dreyer-Alster, S.; Miron, S.; Shirbint, E.; Magalashvili, D.; Flechter, S.; Givon, U.; et al. COVID-19 Vaccination in Patients with Multiple Sclerosis: What We Have Learnt by February 2021. *Mult Scler* **2021**, *27*, 864–870, doi:10.1177/13524585211003476.
