# Supplementary material for: Frequency and Predictors of Relapses following SARS-CoV-2 Vaccination in Patients with Multiple Sclerosis: Interim Results from a Longitudinal Observational Study
Source: J Clin Med. 2023 May 24;12(11):3640. doi: 10.3390/jcm12113640 (PMC10254005; doi:10.3390/jcm12113640)
Supplement: Supplementary file 1 [file jcm-12-03640-s001.zip › REV_Supp Doc S7.pdf]

Supplementary Document S7. Generalizability of patient cohort composition

The cohort of people with multiple sclerosis (PwMS) who participated in this study corresponds to the expected composition of PwMS in Germany regarding socio-demographic and clinical parameters, except for the gender ratio [1,2]. The discrete excess of females in the present study (77.9%) compared with the gender ratio in the German MS Registry (between 71% and 72%), which was known from several studies [1,2], could be due to the current study design (voluntary participation in a structured survey) and has no fundamental impact on the main conclusions. The proportion of patients who reported to receive no disease-modifying drug was also representative as indicated by the analyses of two large German MS patient cohorts [1,3].

References

1. Flachenecker, P.; Eichstädt, K.; Berger, K.; Ellenberger, D.; Friede, T.; Haas, J.; Kleinschnitz, C.; Pöhlau, D.; Rienhoff, O.; Stahmann, A.; et al. [Multiple sclerosis in Germany: updated analysis of the German MS Registry 2014-2018]. *Fortschr Neurol Psychiatr* **2020**, *88*, 436–450, doi:10.1055/a-1248-5258.
2. Ohle, L.-M.; Ellenberger, D.; Flachenecker, P.; Friede, T.; Haas, J.; Hellwig, K.; Parciak, T.; Warnke, C.; Paul, F.; Zettl, U.K.; et al. Chances and Challenges of a Long-Term Data Repository in Multiple Sclerosis: 20th Birthday of the German MS Registry. *Sci Rep* **2021**, *11*, 13340, doi:10.1038/s41598-021-92722-x.
3. Ellenberger, D.; Gisevius, B.; Motte, J.; Flachenecker, P.; Gold, R.; Haas, J.; Hellwig, K.; Timmesfeld, N.; Wiendl, H.; Zettl, U.K.; et al. MS Treatment Profiles in Germany Insights from Two Major MS Cohorts. ECTRIMS, Amsterdam, Netherlands, October 2021.
