# Supplementary material for: Frequency and Predictors of Relapses following SARS-CoV-2 Vaccination in Patients with Multiple Sclerosis: Interim Results from a Longitudinal Observational Study
Source: J Clin Med. 2023 May 24;12(11):3640. doi: 10.3390/jcm12113640 (PMC10254005; doi:10.3390/jcm12113640)
Supplement: Supplementary file 1 [file jcm-12-03640-s001.zip › REV_Supp Figure S1.pdf]

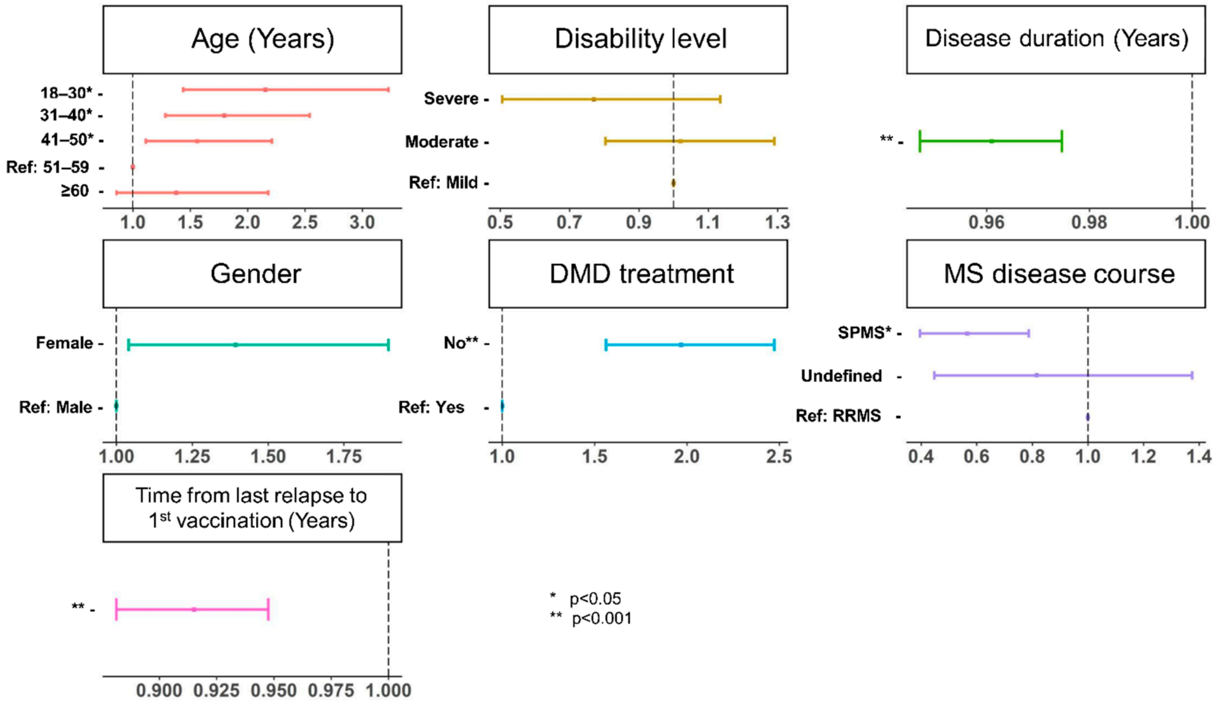

Supplementary Figure S1. Patient characteristics associated with the occurrence of relapses in a univariable logistic regression model. The odds ratios are displayed as dots parallel to the x-axis, flanked by whiskers that define the 95% confidence interval. Post-vaccination relapses were significantly associated with age (OR=0.98, 95% CI: 0.97–0.99, p=0.001), the time from the last relapse (before X^1) to X^1 (OR=0.92, 95% CI: 0.88–0.95, p<0.001), the absence of a DMD treatment (OR=1.97, 95% CI: 1.56–2.47, p<0.001), an RRMS course (OR=1.77, 95% CI: 1.27–2.53, reference: SPMS) and disease duration in the univariable model. CI – confidence interval; DMD – disease-modifying drug; MS – multiple sclerosis; OR – odds ratio; p – p-value; ref – reference; RRMS – relapsing-remitting MS; SPMS – secondary progressive MS.
