# Supplementary material for: Frequency and Predictors of Relapses following SARS-CoV-2 Vaccination in Patients with Multiple Sclerosis: Interim Results from a Longitudinal Observational Study
Source: J Clin Med. 2023 May 24;12(11):3640. doi: 10.3390/jcm12113640 (PMC10254005; doi:10.3390/jcm12113640)
Supplement: Supplementary file 1 [file jcm-12-03640-s001.zip › REV_Supp Table S1.pdf]

Supplementary Table S1. Loss of patients across follow-ups

	N
**Patients at baseline**	2661
**Loss from the baseline to the 1st follow-up**	466
Patients without an invitation (insufficient vaccination interval until the end of the observation period)	2
Patients with complete vaccination status after the first vaccination with Ad26.COV2.S	22
Patients with only one vaccination after SARS-CoV-2 infections	66
Patients with SARS-CoV-2 infections after the first vaccination	4
Patients without specific reasons for non-participation after the baseline survey	372
**Loss from the 1st to the 2nd follow-up**	317
Patients without an invitation (<3 months until the end of the observation period)	112
Patients without specific reasons for non-participation after the 1st follow-up	205

N – number of patients; SARS-CoV-2 – severe acute respiratory syndrome coronavirus 2.
