# Supplementary material for: Frequency and Predictors of Relapses following SARS-CoV-2 Vaccination in Patients with Multiple Sclerosis: Interim Results from a Longitudinal Observational Study
Source: J Clin Med. 2023 May 24;12(11):3640. doi: 10.3390/jcm12113640 (PMC10254005; doi:10.3390/jcm12113640)
Supplement: Supplementary file 1 [file jcm-12-03640-s001.zip › REV_Supp Table S2.pdf]

Supplementary Table S2. Matched pre-pandemic reference registry cohort from 2020 (N=2182)

**Gender**, N (%)	
Female	1724 (79.0)
Male	458 (21.0)
**Age at MS onset [years]**, median (range)	30.0 (24.0–37.3)
**Disease duration [years]**, median (range)	13.3 (6.8–21.3)
**Time to diagnosis [years]**, median (range)	0.3 (0.0–2.2)
**MS disease course**, N (%)	
RRMS	1773 (81.3)
SPMS	409 (18.7)
**Disability level (EDSS)**, N (%)	
Mild (0–2.5)	1168 (53.5)
Moderate (3.0–5.5)	730 (33.5)
Severe (≥6.0)	284 (13.0)
**DMD treatment**, N (%)	
High-efficacy DMD	659 (30.2)
Mild-moderate-efficacy DMD	960 (44.0)
DMD-untreated	563 (25.8)

DMD – disease-modifying drug
EDSS – expanded disability status scale
MS – multiple sclerosis
N – number of patients
RRMS – relapsing remitting MS
SPMS – secondary progressive MS
