# Supplementary material for: Frequency and Predictors of Relapses following SARS-CoV-2 Vaccination in Patients with Multiple Sclerosis: Interim Results from a Longitudinal Observational Study
Source: J Clin Med. 2023 May 24;12(11):3640. doi: 10.3390/jcm12113640 (PMC10254005; doi:10.3390/jcm12113640)
Supplement: Supplementary file 1 [file jcm-12-03640-s001.zip › REV_Supp Table S3.pdf]

Supplementary Table S3. Reference registry cohort of vaccinated MS patients (N=615)

**Gender**, N (%)	
Female	460 (74.8)
Male	155 (25.2)
**Age at MS onset [years]**, median (range)	30.2 (23.2–38.9)
**Disease duration [years]**, median (range)	12.3 (6.4–19.6)
**Time to diagnosis [years]**, median (range)	0.2 (0.0–1.9)
**MS disease course**, N (%)	
RRMS	555 (90.2)
SPMS	60 (9.8)
**Disability level (EDSS)**, N (%)	
Mild (0–2.5)	371 (60.3)
Moderate (3.0–5.5)	168 (27.3)
Severe (≥6.0)	76 (12.4)
**DMD treatment**, N (%)	
High-efficacy DMD	374 (60.8)
Mild-moderate-efficacy DMD	185 (30.1)
Other DMD	3 (0.5)
DMD-untreated	53 (8.6)
**Relapse within the year prior to X^1**, N (%)	50 (8.1)
**Time from last relapse (before X^1) to X^1 [years]**, median (range)	2.1 (1.0–3.3)

DMD – disease-modifying drug
EDSS – expanded disability status scale
MS – multiple sclerosis
N – number of patients
RRMS – relapsing remitting MS
SPMS – secondary progressive MS
X^1 – first SARS-CoV-2 vaccination
